# Delirium risk stratification in consecutive unselected admissions to acute medicine: validation of a susceptibility score based on factors identified externally in pooled data for use at entry to the acute care pathway

**DOI:** 10.1093/ageing/afw198

**Published:** 2016-11-04

**Authors:** Sarah T. Pendlebury, Nicola G. Lovett, Sarah C. Smith, Rose Wharton, Peter M. Rothwell

**Affiliations:** 1 NIHR Oxford Biomedical Research Centre, John Radcliffe Hospital, The University of Oxford, Oxford, UK; 2 Departments of General (Internal) Medicine and Geratology, John Radcliffe Hospital, The University of Oxford, Oxford, UK; 3 Stroke Prevention Research Unit, Nuffield Department of Clinical Neurosciences, John Radcliffe Hospital, The University of Oxford, Oxford, UK

**Keywords:** delirium, prediction, risk score, external validation, acute medicine, older people

## Abstract

**Background:**

recognition of prevalent delirium and prediction of incident delirium may be difficult at first assessment. We therefore aimed to validate a pragmatic delirium susceptibility (for any, prevalent and incident delirium) score for use in front-line clinical practice in a consecutive cohort of older acute medicine patients.

**Methods:**

consecutive patients aged ≥65 years over two 8-week periods (2010–12) were screened prospectively for delirium using the Confusion Assessment Method (CAM), and delirium was diagnosed using the DSM IV criteria. The delirium susceptibility score was the sum of weighted risk factors derived using pooled data from UK-NICE guidelines: age >80 = 2, cognitive impairment (cognitive score below cut-off/dementia) = 2, severe illness (systemic inflammatory response syndrome) = 1, infection = 1, visual impairment = 1. Score reliability was determined by the area under the receiver operating curve (AUC).

**Results:**

among 308 consecutive patients aged ≥65 years (mean age/SD = 81/8 years, 164 (54%) female), AUC was 0.78 (95% CI 0.71–0.84) for any delirium; 0.71 (0.64–0.79), for prevalent delirium; 0.81 (0.70–0.92), for incident delirium; odds ratios (ORs) for risk score 5–7 versus <2 were 17.9 (5.4–60.0), *P* < 0.0001 for any delirium, 8.1 (2.2–29.7), *P* = 0.002 for prevalent delirium, and 25.0 (3.0–208.9) *P* = 0.003 for incident delirium, with corresponding relative risks of 5.4, 4.7 and 13. Higher risk scores were associated with frailty markers, increased care needs and poor outcomes.

**Conclusions:**

the externally derived delirium susceptibility score reliably identified prevalent and incident delirium using clinical data routinely available at initial patient assessment and might therefore aid recognition of vulnerability in acute medical admissions early in the acute care pathway.

## Introduction

Effective delirium management requires recognition of prevalent delirium and identification of those at future risk to guide individualised patient care including targeted multicomponent interventions [1–3]. However, recognition of prevalent delirium at initial patient assessment may be difficult owing to lack of available informant or because the fluctuating nature of the condition means that a period of observation is required: establishing the time course of behavioural change is a key component of validated screening tools such as the Confusion Assessment Method (CAM) [4] and the 4AT [5]. Predicting delirium risk may also be difficult in individual patients [6]. Fragmented care, acute care workload and lack of continuity bring additional challenges.

A score to identify risk of any delirium that is present at first assessment (“prevalent”) as well as occurring during admission (“incident”) delirium would therefore be helpful in enabling recognition of a vulnerable group with high care needs at the earliest point in the care pathway particularly in busy clinical settings and would facilitate selection of appropriate care in the absence of a definite delirium diagnosis [1, 2]. Such a score would need to be pragmatic, simple and to use only routinely collected clinical data available at first assessment. We have previously examined existing delirium risk scores in older patients in acute general medicine [7], but these scores used factors obtained from single-institution–derived data sets, required simplification from their original published forms and reliability was only moderate.

We therefore aimed to validate a new delirium susceptibility score based on the risk factors identified in pooled data from UK-NICE guidelines [1] available at the point of initial patient assessment. The score was designed to function as both a diagnostic (cross-sectional) and prognostic (longitudinal) model [6] to predict susceptibility to both prevalent and incident (any) delirium. We examined the reliability of the susceptibility score in a consecutive, inclusive and representative cohort of older acute medicine patients for any, prevalent and incident delirium and compared it to existing scores examined in the previous study. Finally, we determined the ‘face validity’ of the score through examining the relationship between delirium susceptibility as defined by the score and associates of delirium including markers of frailty, high care needs and poor outcomes.

## Methods

### Patient cohort

The Oxford University Hospitals Trust (OUHT) provides secondary care services for a population of approximately 500,000. In a prospective observational audit, consecutive unselected admissions to the acute medicine team over two 8-week periods (September to November 2010 and April to June 2012) were screened for delirium on arrival and thereafter until discharge, transfer or death. The audit was undertaken to inform future service development and was approved by the Divisional Management (audit registration Datix 2197). All data were routinely acquired as part of standard patient care. Data on age-specific delirium rates and outcomes from this cohort together with external validation of existing delirium risk scores have been published previously [7, 8].

Only patients aged ≥65 years were included in the current study. The methodology for patient assessment and delirium diagnosis has been described previously [7, 8]. Briefly, all patients were seen within 24 hours of admission and managed by the Consultant Physician (S.T.P., S.C.S.) responsible for the patient's care. All patients had a validated cognitive screen as part of the standard OUHT clerking proforma [9], which included the CAM [4] and a cognitive test (Mini-Mental State Examination (MMSE) [10] or Abbreviated Mental Test Score (AMTS)) [11]. Delirium diagnosis was made according to DSM IV criteria [12] by the responsible physician (S.T.P., S.C.S.) after discussion with the rest of the medical team and was categorised as prevalent delirium (on admission or within the first 48 hours), incident delirium (occurring after the first 48 hours) or any delirium (occurring at any point during admission).

Demographic and clinical data were recorded from the patient, relatives and primary care physician (general practitioner (GP)) and medical records. The Charlson index for co-morbidities was calculated for all patients [13]. The Malnutrition Universal Screening Tool (MUST, at risk ≥1) [14] and Pressure Sore Prediction Score (PSPS, at risk ≥6) [15] for pressure area vulnerability were routinely recorded by nursing staff. Urinary or faecal incontinence, falls, constipation requiring intervention (new laxative prescription or bowel care) and sleep deprivation were documented prospectively. Length of stay was calculated for the time spent in the acute hospital. Increased care needs at discharge were defined as new placement or new or increased level of care package at home or discharge to community hospital for rehabilitation.

### Delirium susceptibility score

The susceptibility score was designed to predict risk of any, i.e. both prevalent and incident delirium at initial patient assessment in the acute care setting in line with the Transparent Reporting of a multivariable prediction model for Individual Prognosis Or Diagnosis (TRIPOD) guidelines [6, 16]. Factors reported in the UK-NICE guidelines from pooled meta-analyses as independently associated with delirium and available at the point of initial assessment were selected for use in the score (dementia/cognitive impairment, age ≥80 years, severe illness, infection and visual impairment) (Table 1) [1, 6]. We did not consider factors that were possibly associated with delirium (co-morbidity, polypharmacy, dehydration (blood urea nitrogen: creatinine ratio), electrolyte disturbance, depression) or factors occurring during admission (bladder catheter insertion) or specific to specialist settings (hip fracture) [1]. The risk score was generated for each patient by assigning numeric values of 1 of 2 according to the strength of association reported in the guidelines (maximum score = 7, Table 1).

**Table 1.** Derivation of the delirium susceptibility score using systematically reviewed pooled data reported in the UK-NICE guidelines

Factor reported in NICE guideline [1]	Strength of reported association OR, 95% CI	Routinely available data used in the risk score	Allocated weight
Dementia/cognitive impairment	6.3, 2.9–13.7	Known diagnosis of dementia and or cognitive score below cut-off (AMTS < 9 or MMSE < 24)	2
Age ≥80 years	5.2, 2.6–10.4	Age	2
Severe illness	3.5, 1.5–8.2	Systemic inflammatory response syndrome (SIRS) positive^1^	1
Infection	3.0, 1.4–6.2	Working diagnosis of infection	1
Vision impairment	1.7, 1.0–2.9	History of poor vision in the care record or clinically overt poor vision	1

^1^SIRS was classed as positive if 2 or more of the following were present: heart rate >90 beats per minute, temperature <36°C or >38°C, respiratory rate >20 breaths per minute, white blood cell count <4 × 10^9^ or >12 × 10^9^ cells per litre [17].

Risk factors were defined pragmatically to ensure clinical feasibility. Dementia/cognitive impairment was defined as a known diagnosis of dementia or a cognitive score below cut-off (MMSE < 24 or AMTS < 9) as described previously [7–9]. Severe illness was defined by presence of the systemic inflammatory response syndrome (SIRS) since this could be derived using only data routinely acquired at initial patient assessment and was classed as positive if two or more of the following were present: heart rate >90 beats per minute, temperature <36°C or >38°C, respiratory rate >20 breaths per minute, white blood cell count <4 × 10^9^ or >12 × 10^9^ cells per litre [17]. Vision impairment was recorded if noted in the medical history or was evident during patient admission.

### Statistical analyses and risk score validation

Reliability of the score for any, prevalent and incident delirium in our cohort was established using the area under the receiver operating characteristic curve (AUC). To determine the performance of the score for identifying risk of prevalent delirium, all patients were included. For analyses of incident delirium, patients with prevalent delirium were excluded. Missing data were not imputed except for cognitive data where AUCs were calculated both without and with imputed data with missing scores imputed as normal. Sensitivity, specificity, positive and negative predictive values were calculated. Statistical differences between the AUCs obtained for the new score and existing scores were tested with pairwise comparisons using the *z* test with correction for multiple (*n* = 4) comparisons such that *P* < 0.013 was significant.

Sensitivity analyses were performed for AUCs without differential weighting of the risk score factors (i.e. all factors allocated a score of 1) and after exclusion of each factor in turn. Reliability of the model was also determined after addition of each of the two factors (functional dependency, defined as residence in a care home or at home with carers, and clinical dehydration) contained in existing acute medicine models validated in our data set [7] but not included in the new model.

To determine the ‘face validity’ or information content of the risk score [6], odds ratios (ORs) were calculated for univariable associations between clinical factors including known associates of delirium not included in the score and tertiles of delirium risk (≤1, 2–4, 5–7), unadjusted and adjusted for age.

## Results

Three hundred and eight consecutive patients aged ≥65 years (mean/SD age 81/8 years, 164 (54%) female) were admitted by our acute medicine team over the 4-month period. Any delirium occurred in 95 patients (31%) (67 with prevalent delirium of whom 17 had recurrent episodes and 28 with incident delirium). Rates of missing data for parameters required for score completion were generally low (SIRS *n* = 3, infection *n* = 7, age *n* = 0, visual impairment *n* = 14) except for cognitive test in patients without prior dementia (*n* = 79 no reason documented, *n* = 12 too unwell, *n* = 3 dysphasic, *n* = 1 no English).

AUCs for the susceptibility score were 0.78 (95% CI 0.71–0.84) for any, 0.71 (95% CI 0.64–0.79) for prevalent and 0.81 (95% CI 0.70–0.92) for incident delirium with no major differences after weighting all factors equally (Table 2). Imputation of missing cognitive data made little difference to the overall AUC for any delirium (0.77, 95% CI 0.71–0.82) but improved AUC for prevalent delirium (0.74, 95% CI 0.68–0.81) at the expense of incident delirium (0.74, 95% CI 0.63–0.85, Appendix Table 1). The susceptibility score had higher AUC for any delirium than any of the other published risk scores previously validated in our cohort, but AUCs were broadly similar with little significant difference after correction for multiple comparisons (Appendix Table 2 and Figure). When cognitive impairment, infection and severe illness defined by SIRS were removed in turn from the model, AUCs were non-significantly lower suggesting that all these factors contributed to the model. However, removal of the visual impairment factor had no effect, whereas removal of the older age (>80 years) factor resulted in an increase in AUC values: 0.80 (0.74–0.86) for any, 0.74 (0.67–0.81) for prevalent and 0.84 (0.77–0.92) for incident delirium (Table 2).

**Table 2.** AUCs for the delirium susceptibility score for any, prevalent and incident delirium

	AUC		
	Any *n* = 205	Prevalent *n* = 205	Incident *n* = 150
Weighted score	0.78, 0.71–0.84	0.71, 0.64–0.79	0.81, 0.70–0.92
Unweighted score	0.78, 0.72–0.85	0.73, 0.66–0.80	0.79, 0.69–0.90
After removal of individual factors from the weighted model			
Without visual impairment	0.77, 0.71–0.84	0.71, 0.64–0.79	0.81, 0.70–0.92
Without cognitive impairment	0.70, 0.63–0.78	0.66, 0.59–0.75	0.72, 0.59–0.84
Without infection	0.72, 0.65–0.79	0.66, 0.58–0.74	0.77, 0.66–0.88
Without age	0.80, 0.74–0.86	0.74, 0.67–0.81	0.84, 0.77–0.92
Without SIRS	0.76, 0.69–0.82	0.69, 0.62–0.77	0.72, 0.59–0.84
After addition of other factors contained in existing models to the weighted model			
With clinical dehydration	0.78, 0.65–0.80	0.73, 0.65–0.80	0.80, 0.69–0.91
With functional impairment	0.76, 0.70–0.83	0.72, 0.64–0.79	0.78, 0.67–0.89

AUCs are shown for both weighted and unweighted models and for the weighted model after removal of each factor in the model in turn and after the addition of other factors contained in existing models.

Appendix Table 3 shows the sensitivities, specificities, positive and negative predictive values for the susceptibility score for any, prevalent and incident delirium. ORs for risk score 5–7 versus <2 were 17.9 (5.4–60.0) *P* < 0.0001 for any delirium, 8.1 (2.2–29.7) *P* = 0.002 for prevalent delirium and 25.0 (3.0–208.9) *P* = 0.003 for incident delirium. Only 4/30 (13%) patients with scores <2 had any delirium versus 43/58 (74%) with scores of 5–7 giving a relative risk (RR) of 5.4 for the highest versus the lowest tertile of risk with higher RR for incident delirium (Appendix Table 3).

Factors strongly associated (*P* < 0.0001) with increasing tertiles of delirium risk score were previous history of falls (OR = 3.0, 1.7–5.4), prior TIA/stroke (OR = 3.1, 1.7–5.7), functional dependency (OR = 2.2, 1.2–3.9), clinical dehydration (OR = 3.8, 1.9–7.3), urinary (OR = 4.3, 2.4–7.9) and faecal (OR = 4.6, 2.2–9.5) incontinence (Table 3). Less strong associations were seen for pressure sore risk, being bedbound, sleep deprivation, urinary catheter insertion, length of stay, increased care needs on discharge and mortality with trends to inpatient falls and male sex (Table 3).

**Table 3.** Factors not included in the score associated with increasing tertiles of delirium susceptibility score

	Susceptibility score			OR	*P*	OR adj	*P* adj
	≤1, *n* = 70	2–4, *n* = 162	5–7, *n* = 60				
Demographic factors							
Male sex	34	67	30	1.0 (0.7, 1.6)	0.949	1.7 (0.9, 2.5)	0.088
Past medical history							
Falls	10	45	36	4.2 (2.5, 7.1)	<0.0001	**3.0 (1.7, 5.4)**	**<0.0001**
TIA/Stroke	5	39	25	3.5 (2.1, 6.1)	<0.0001	**3.1 (1.7, 5.7)**	**<0.0001**
Depression	14	30	11	0.9 (0.5, 1.6)	0.8	1.0 (0.6, 1.9)	0.925
Charlson > 3	6	24	7	1.2 (0.7, 2.4)	0.521	1.2 (0.6, 2.4)	0.716
Medications > 3	48	131	51	1.9 (1.1, 3.4)	0.022	1.6 (0.8, 2.9)	0.185
Medications > 7	22	67	23	1.2 (0.8, 1.9)	0.408	1.0 (0.6, 1.6)	0.952
Previous dependency							
Care Home/care package	8	41	33	4.3 (2.6, 7.4)	<0.0001	**2.2 (1.2, 3.9)**	**0.008**
Care Home/Comm. Hosp.	1	17	13	3.9 (1.9, 7.9)	<0.0001	1.6 (0.7, 3.6)	0.284
Clinical parameters							
Clinical dehydration	6	27	23	3.5 (1.9, 6.3)	<0.001	**3.8 (1.9, 7.3)**	**<0.0001**
Low oxygen saturation	17	39	19	1.2 (0.7, 2.1)	0.409	1.2 (0.7, 2.0)	0.638
PSPS ≥ 6	10	20	20	3.0 (1.5, 5.9)	0.002	**2.4 (1.2, 5.2)**	**0.02**
MUST > 0	2	12	8	2.9 (1.2, 7.2)	0.021	1.9 (0.7, 5.1)	0.232
During admission							
Urinary incontinence	8	36	33	4.5 (2.6, 7.8)	<0.0001	**4.3 (2.4, 7.9)**	**<0.0001**
Faecal incontinence	7	16	24	4.5 (2.3, 8.6)	<0.0001	**4.6 (2.2, 9.5)**	**<0.0001**
Bedbound	7	27	22	3.1 (1.7, 5.5)	<0.0001	**2.8 (1.5, 5.4)**	**0.002**
Sleep deprivation	5	22	18	3.1 (1.6, 5.8)	<0.001	**3.4 (1.7, 6.8)**	**0.001**
Constipation	5	26	14	2. 2 (1.2, 4.0)	0.014	1.4 (0.7, 2.8)	0.347
Falls	2	7	6	2.5 (0.9, 7.0)	0.073	2.7 (0.9, 7.9)	0.077
CT brain scanning	8	27	9	1.2 (0.7, 2.2)	0.559	1.8 (0.9, 3.6)	0.087
Urinary catheter	4	20	16	3.1 (1.6, 6.0)	0.001	**2.4 (1.2, 5.1)**	**0.017**
Outcome							
Stay > 7days	17	56	36	2.7 (1.7, 4.4)	<0.0001	**2.3 (1.4, 4.0)**	**0.002**
New placement	3	20	7	1.8 (0.9, 3.7)	0.1	1.6 (0.7, 3.6)	0.263
Increased care	6	34	14	2.1 (1.2, 3.7)	0.012	**2.1 (1.1, 4.1)**	**0.022**
Death during admission	3	9	8	2.5 (1.0, 6.2)	0.043	**2.9 (1.1, 7.8)**	**0.03**

Values significant at P < 0.05 after adjustment for age and sex are shown in bold. Adj, adjusted for age and sex.

## Discussion

The proposed delirium susceptibility score, based on risk factors derived externally using pooled data, was reliable in identifying patients at risk of any (both prevalent and incident) delirium with three-quarters of those in the highest tertile affected. Higher scores were also associated with markers of frailty, high care needs and poor outcomes indicating good face validity. The new score performed at least as well as existing scores and contained only factors easily available at initial patient assessment making it practical for use in the acute setting.

In a previous study [7], we examined the reliability of published acute medicine delirium risk scores many of which used non-routinely available data (e.g. detailed questionnaires on functional ability, non-standard cognitive assessments and multidisciplinary assessments of illness severity), which required simplification prior to validation in our data set. Despite the modifications, we found that all scores performed better than chance and all predicted prevalent delirium even when developed to detect incident delirium. The validation was robust by the TRIPOD criteria in using a geographically and institutionally distinct, inclusive and representative data set and different measurements for the various risk factors [6].

The new score was developed using factors reported as independently associated with delirium in pooled analyses from multiple studies [1], whereas existing scores were derived from factors obtained from single data sets. AUCs for the new score were higher but broadly similar to the simplified existing scores although our study was under powered to detect small differences between scores. Reliability was overall less good for prevalent delirium possibly because of the relatively greater importance of on-admission illness severity and infection [8].

Choice of score will depend in part on pragmatic considerations regarding which is easiest to administer, a key criterion for clinical utility [6]. For example, baseline functional impairment, included in several previously published scores, may be difficult to assess in acutely unwell patients particularly before reliable collateral history is obtained [7]. In contrast, the score proposed in the current study contains only items that should be easily available in the majority of patients at initial assessment and, in fact, addition of factors contained in existing models including functional impairment did not improve the AUC of the new score suggesting strong shared associations between factors.

Our data demonstrate that delirium risk stratification of patients at the start of the acute care pathway is feasible and might facilitate early patient management particularly in those without overt prevalent delirium including signposting location of care (e.g. ward with multicomponent intervention capability, delirium expertise). A higher awareness for the potential for delirium might also help recognition of prevalent and incident cases at subsequent review. Management according to overall delirium susceptibility might be particularly helpful in busy or non-specialist clinical settings or where there is lack of continuity of care and possibly also in hospital-at-home or acute ambulatory units in counselling patients and families regarding the likelihood of worsening or fluctuating cognitive function or in predicting need for admission. With the advent of electronic patient records, delirium susceptibility could be calculated using algorithms that could be used to automate individualised care. The cut-offs used to determine targeting of multicomponent interventions would need to be determined locally according to service structure but delirium rates were low in those with scores of ≤1, and thus interventions would appear unjustified in this group.

Strengths of our study include the prospective inclusive cohort design, the pragmatic use of easily available factors routinely collected in the course of the patient's clinical care and the external derivation and validation of the new risk score in line with the TRIPOD guidelines [6]. There are some limitations to our study. First, there was no independent delirium assessment so we were unable to assess the robustness of our delirium diagnoses. However, significant under-recognition was unlikely given that measured delirium rates were in line with previous studies[1,2]. Second, the susceptibility score was designed to combine the functions of a cross-sectional (diagnostic) and longitudinal (prognostic) tool [6]. However, both are ‘prediction’ models differing only in the concept of time [6]. Third, some acutely unwell older patients are not testable using even a simple cognitive test resulting in lack of applicability of the risk score to these patients [9, 18]. Further external validations are required and future studies should consider whether untestability should be classed in the same way as cognitive impairment for the purposes of delirium risk stratification since available data suggest that untestability is associated with illness severity and severe cognitive impairment [18]. Finally, studies are required to evaluate the clinical utility of the score in routine practice and whether it can target interventions in a way that improves patient care.

In conclusion, our findings suggest that the delirium susceptibility score could be used at the earliest point in the acute care pathway to stratify risk of both prevalent and incident delirium and to identify vulnerable groups with high care needs. This would enable early selection of appropriate care pathways in the absence of formal delirium diagnosis, facilitate discussions with patients and families, aid prognostication and could be automated for use with electronic patient records.

Key points

- The susceptibility score was reliable for any (incident and prevalent) delirium.
- The delirium susceptibility score identified those with high care needs, frailty markers and poor outcomes.
- The delirium susceptibility score used only factors routinely available at the earliest point in the acute care pathway.
- Three-quarters of patients with highest tertile scores (5–7) had delirium.
- The delirium susceptibility score had higher AUCs than existing scores in our data set.

## Supplementary Material

Supplementary Data
