# Supplementary material for: Delirium risk stratification in consecutive unselected admissions to acute medicine: validation of a susceptibility score based on factors identified externally in pooled data for use at entry to the acute care pathway
Source: Age Ageing. 2016 Nov 4;46(2):226–31. doi: 10.1093/ageing/afw198 (PMC5386005; doi:10.1093/ageing/afw198)
Supplement: Supplementary Data [file afw198_SUPPLEMENTARY_DATA.docx]

**SUPPLEMENTARY DATA**

Appendix Table 1. AUCs for the delirium susceptibility score for any, prevalent and incident delirium after imputation of missing cognitive data. AUCs are shown for both weighted and unweighted models and for the weighted model after removal of each factor in the model in turn and after the addition of other factors contained in existing models.

| AUC, with imputation of missing cognitive data | | | | | | | |
| --- | --- | --- | --- | --- | --- | --- | --- |
|  | Any  n=292 | | Prevalent  n=292 | | | Incident  n=227 | |
| Weighted score | 0.77, 0.71-0.82 | | 0.74, 0.68-0.81 | | | 0.74, 0.63-0.85 | |
| Unweighted score | 0.77, 0.71-0.82 | | 0.76, 0.69-0.82 | | | 0.71, 0.61-0.82 | |
| After removal of individual factors from the model | | | | | | | |
| Without visual impairment | 0.77, 0.71-0.82 | | | 0.75, 0.68-0.81 | | | 0.73, 0.62-0.84 |
| Without cognitive impairment | 0.70, 0.64-0.77 | | | 0.69, 0.62-0.76 | | | 0.68, 0.57-0.78 |
| Without infection | 0.73, 0.67-0.79 | | | 0.70, 0.63-0.77 | | | 0.73, 0.63-0.83 |
| Without age | 0.77, 0.72-0.83 | | | 0.76, 0.69-0.82 | | | 0.74, 0.62-0.85 |
| Without SIRS | 0.76, 0.70-0.82 | | | 0.73, 0.66-0.79 | | | 0.76, 0.66-0.85 |
| After addition of other factors contained in existing models to the weighted model | | | | | | | |
| With clinical dehydration | | 0.78, 0.72-0.84 | | | 0.76, 0.70-0.82 | | 0.75, 0.64-0.85 |
| With functional impairment | | 0.75, 0.69-0.81 | | | 0.74, 0.67-0.81 | | 0.70, 0.59-0.81 |

Appendix Table 2. AUC for any, prevalent and incident delirium for existing delirium risk scores in acute medicine [1] and Z test for significance of difference between existing scores tested pairwise against the new score.

| AUC, 95% CI, delirium; p values versus AUC for the new score | | | | | | |
| --- | --- | --- | --- | --- | --- | --- |
|  | Any | p | Prevalent | p | Incident | p |
| Inouye et al^2^ | 0.73 (0.66, 0.80) | 0.08 | 0.70 (0.62, 0.78) | 0.68 | 0.73 (0.63, 0.83) | 0.042 |
| Martinez et al^3^ | 0.69 (0.62, 0.76) | 0.002 | 0.61 (0.53, 0.69) | 0.001 | 0.78 (0.68, 0.87) | 0.43 |
| Isfandiaty et al^4^ | 0.76 (0.70, 0.83) | 0.58 | 0.69 (0.61, 0.77) | 0.41 | 0.82 (0.74, 0.91) | 0.77 |
| Douglas et al^5^ | 0.74 (0.67, 0.81) | 0.01 | 0.68 (0.60, 0.76) | 0.046 | 0.78 (0.68, 0.88) | 0.27 |

References

1. Pendlebury ST, Lovett N, Smith SC, Cornish E, Mehta Z, Rothwell PM. Delirium risk stratification in consecutive unselected admissions to acute medicine: validation of externally derived risk scores. Age Ageing. 2016;45:60-5.

2. Inouye SK, Viscoli CM, Horwitz RI, Hurst LD, Tinetti ME. A predictive model for delirium in hospitalized elderly medical patients based on admission characteristics. Ann Intern Med. 1993;119:474-81.

3. Martinez JA, Belastegui A, Basabe I, et al. Derivation and validation of a clinical prediction rule for delirium in patients admitted to a medical ward: an observational study. BMJ Open.2012;2(5).

4. Isfandiaty R, Harimurti K, Setiati S, Roosheroe AG. Incidence and predictors for delirium in hospitalized elderly patients: a retrospective cohort study. Acta Med Indones. 2012;44:290-7.

5. Douglas VC, Hessler CS, Dhaliwal G, et al. The AWOL tool: derivation and validation of a delirium prediction rule. J Hosp Med. 2013;8:493-9.


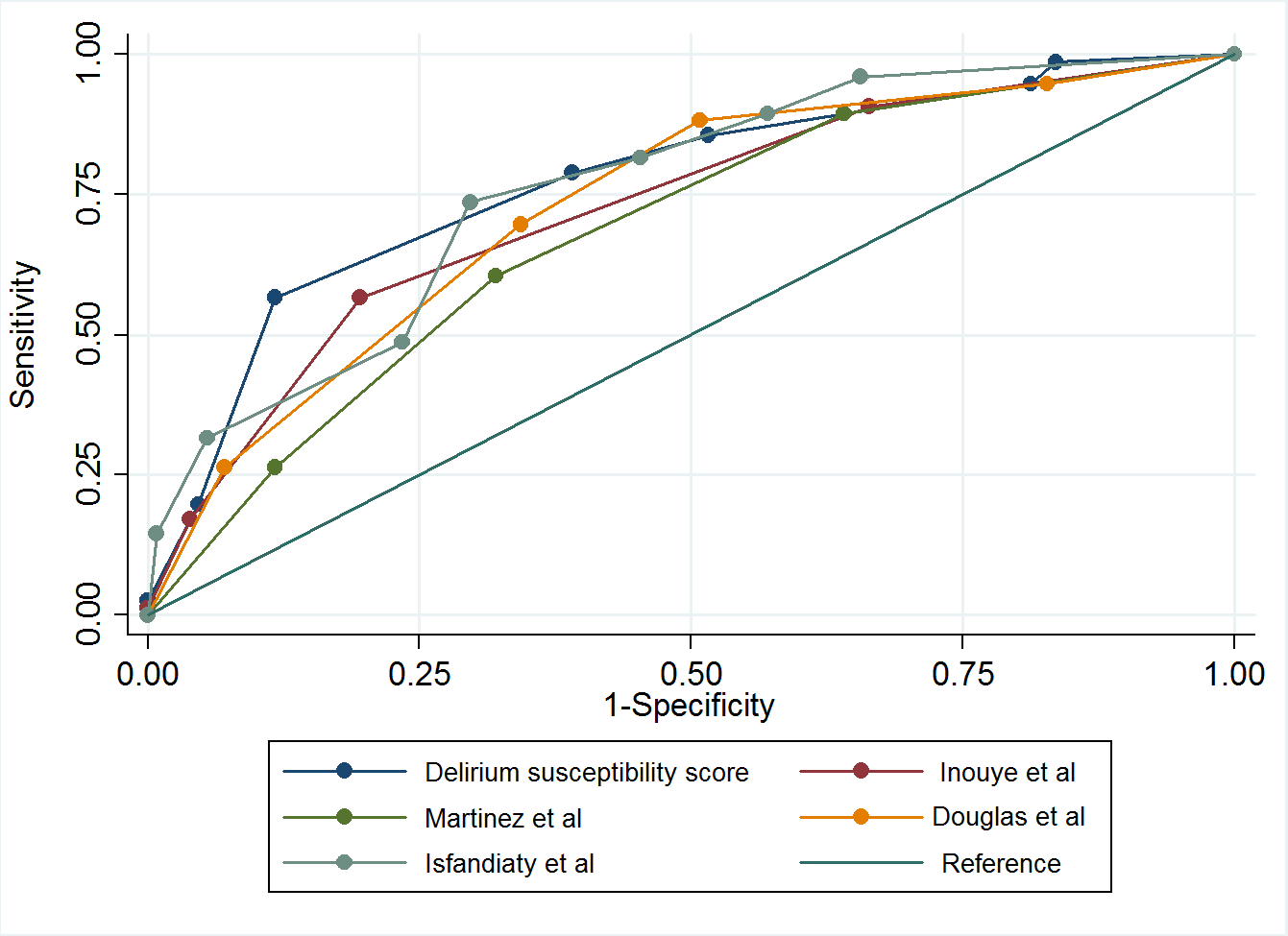


Appendix Figure

Appendix Table 3. Sensitivity and specificity, positive and negative predictive values for the risk score and OR and RR for each risk score category versus the lowest risk category (≤1).

| Risk score | Sensitivity | Specificity | PPV | NPV | |  |
| --- | --- | --- | --- | --- | --- | --- |
| Any delirium | | | | | | |
| 0 |  |  |  | |  | |
| 1 | 0.99 | 0.17 | 0.41 | | 0.96 | |
| 2 | 0.95 | 0.19 | 0.41 | | 0.86 | |
| 3 | 0.86 | 0.49 | 0.5 | | 0.85 | |
| 4 | 0.79 | 0.61 | 0.55 | | 0.83 | |
| 5 | 0.57 | 0.88 | 0.74 | | 0.78 | |
| 6 | 0.2 | 0.95 | 0.71 | | 0.67 | |
| 7 | 0.03 | 1 | 1 | | 0.64 | |
| Risk score | **OR** | **95 % CI** | **p** | | **RR** | |
| <= 1 | (1.0) |  |  | |  | |
| 2-4 | 2.04 | 0.65, 6.34 | 0.22 | | 1.78 | |
| 5-7 | 17.92 | 5.35, 59.97 | <0.0001 | | 5.38 | |
|  |  |  |  | |  | |
| Prevalent delirium | | | | | | |
| 0 |  |  |  | |  | |
| 1 | 0.98 | 0.15 | 0.3 | | 0.96 | |
| 2 | 0.95 | 0.17 | 0.3 | | 0.9 | |
| 3 | 0.85 | 0.44 | 0.36 | | 0.89 | |
| 4 | 0.78 | 0.55 | 0.39 | | 0.87 | |
| 5 | 0.51 | 0.8 | 0.48 | | 0.82 | |
| 6 | 0.16 | 0.92 | 0.43 | | 0.75 | |
| 7 | 0.04 | 1 | 1 | | 0.74 | |
| Risk score | **OR** | **95 % CI** | **p** | | **RR** | |
| <= 1 | (1.0) |  |  | |  | |
| 2-4 | 2.21 | 0.62, 7.93 | 0.223 | | 1.97 | |
| 5-7 | 8.09 | 2.20, 29.72 | 0.002 | | 4.67 | |
|  | | | | | | |
| Incident delirium | | | | | | |
| 0 |  |  |  | |  | |
| 1 | 1 | 0.17 | 0.16 | | 1 | |
| 2 | 0.95 | 0.19 | 0.16 | | 0.96 | |
| 3 | 0.86 | 0.49 | 0.21 | | 0.95 | |
| 4 | 0.81 | 0.61 | 0.25 | | 0.95 | |
| 5 | 0.71 | 0.88 | 0.5 | | 0.95 | |
| 6 | 0.29 | 0.95 | 0.5 | | 0.89 | |
| 7 | 0 | 1 | 1 | | 0.86 | |
| Risk score | **OR** | **95 % CI** | **p** | | **RR** | |
| <= 1 | (1.0) |  |  | |  | |
| 2-4 | 1.4 | 0.16, 12.58 | 0.761 | | 1.38 | |
| 5-7 | 25 | 2.99, 208.91 | 0.003 | | 13 | |
